# A unique intra-molecular fidelity-modulating mechanism identified in a viral RNA-dependent RNA polymerase

**DOI:** 10.1093/nar/gky848

**Published:** 2018-09-20

**Authors:** Weichi Liu, Xiaoling Shi, Peng Gong

**Affiliations:** 1 Key Laboratory of Special Pathogens and Biosafety, Wuhan Institute of Virology, Chinese Academy of Sciences, Wuhan, Hubei 430071, China; 2 University of Chinese Academy of Sciences, Beijing 100049, China

## Abstract

Typically not assisted by proofreading, the RNA-dependent RNA polymerases (RdRPs) encoded by the RNA viruses may need to independently control its fidelity to fulfill virus viability and fitness. However, the precise mechanism by which the RdRP maintains its optimal fidelity level remains largely elusive. By solving 2.1–2.5 Å resolution crystal structures of the classical swine fever virus (CSFV) NS5B, an RdRP with a unique naturally fused N-terminal domain (NTD), we identified high-resolution intra-molecular interactions between the NTD and the RdRP palm domain. In order to dissect possible regulatory functions of NTD, we designed mutations at residues Y471 and E472 to perturb key interactions at the NTD–RdRP interface. When crystallized, some of these NS5B interface mutants maintained the interface, while the others adopted an ‘open’ conformation that no longer retained the intra-molecular interactions. Data from multiple *in vitro* RdRP assays indicated that the perturbation of the NTD–RdRP interactions clearly reduced the fidelity level of the RNA synthesis, while the processivity of the NS5B elongation complex was not affected. Collectively, our work demonstrates an explicit and unique mode of polymerase fidelity modulation and provides a vivid example of co-evolution in multi-domain enzymes.

## INTRODUCTION

Processive nucleic acid polymerases are essential for the preservation, passage, and evolution of the genetic information. Optimal fidelity levels of processive polymerase synthesis, in some cases coupled to proofreading and/or repairing processes carried out by polymerase itself or other machineries (1–4), are critical for nearly all forms of life. The RNA viruses are a large and unique group of species whose genetic information are solely carried in the form of RNA, and the related genome replication process is dependent on the virally encoded RNA-dependent RNA polymerase (RdRP) and typically not assisted by proofreading mechanisms (5,6). Well known as quasi-species, the RNA viruses undergo relatively rapid evolution and exist as populations bearing genome-wide distributed mutations (7–9). It was proposed that the RNA viruses live with a narrow but optimal range of replication error frequency, as higher error rates may lead to distinction of the species and lower error rates may fail to overcome selection pressure (10). As primary machineries that contribute to the replication error of the RNA viruses, viral RdRPs are unique systems for understanding how optimal fidelity is achieved.

Viral RdRPs all contain a catalytic core that is analogous to an encircled human right hand comprising palm, fingers and thumb domains (11–13). Seven catalytic motifs A–G surround the active site with A–E in the most conserved palm and F/G in the fingers (14–16). The encirclement created by the interactions between the finger tips and thumb makes the distinction from other right-hand polymerases such as the klenow fragment of DNA polymerase I, the bacteriophage T7 RNA polymerase and the human immunodeficiency virus 1 reverse transcriptase (HIV-1 RT) (17–19). Accordingly, the fingers-thumb interactions in the RdRP may restrict large-scale fingers domain conformational changes typically seen in other polymerases (20–22). Indeed, relative local rearrangement in the palm domain is responsible for the active site closure in viral RdRP nucleotide addition cycle (23). This rearrangement primarily involves coordinated backbone movement of motifs A and D and key side chain rotamer changes within motifs A, B and F (23–26).

As the major determinants of polymerase fidelity, the NTP-binding induced pre-chemistry active site closure is the key process for fidelity modulation (27). To date, fidelity variants both in the levels of the RdRP and the full-length virus have been identified through approaches including RdRP structure-based rational mutation design and virus fidelity variant screening (28–33). Somewhat unexpectedly, the variation/mutation sites have been found widely distributed in the RdRP core, not limited to the aforementioned catalytic motifs or key residues known to participate in active site closure. Some of the mutations were believed to modulate fidelity through indirect interactions with or long-range transmission to the active site (34,35). It was suggested that the mutations in the RdRP palm domain, where the majority of the conformational changes occur during the active site closure, had greater impact on fidelity than those in the fingers domain had (31). However, the precise mechanism by which each mutation/variation modulates fidelity and whether and how fidelity can be directionally controlled by engineering for purposes including attenuated vaccine development remain poorly understood (36,37).

The pestiviruses, including classical swine fever virus (CSFV) and bovine viral diarrhea virus (BVDV), are a small group of livestock pathogens belonging to the *Pestivirus* genus and *Flaviviridae* family and their RdRPs were given the name of NS5B. Compared to the RdRPs of other *Flaviviridae* representatives such as the NS5 of the Japanese encephalitis virus (JEV) and dengue virus (DENV) in the *Flavivirus* genus and the NS5B of the hepatitis C virus (HCV) of the *Hepacivirus* genus, the pestivirus NS5B contains a unique ∼90-residue N-terminal domain (NTD) that does not have notable sequence homology to any other viral or host proteins. Previous determined pestivirus NS5B crystal structures (of bovine viral diarrhea virus, or BVDV) do not include the NTD (38,39), and show a global architecture similar to the RdRP module of HCV NS5B and flavivirus NS5 (11,40,41). A very recent work reported an NTD-containing CSFV (Eystrup strain) NS5B crystal structure of moderate resolution (3.0 Å), revealing the overall fold and the intra-molecular interactions between the NTD and the RdRP module (42). Functional studies have implied that the NTD contributes to the polymerase activity (42–44), but the precise mechanism of how the NTD regulates polymerase catalysis remains elusive.

In this work, by solving three high-resolution (up to 2.1 Å) crystal structures of the NTD-containing CSFV (Shimen Strain) NS5B, we provide detailed structural information of the NTD and its intra-molecular interactions with the RdRP core. Bearing a unique α/β fold, the NTD interacts with the RdRP palm domain in the vicinity of motifs A and D. Crystallographic and enzymatic characterizations of CSFV NS5B and its NTD–RdRP interface mutants further demonstrated that NTD contributed to the optimal fidelity of the RdRP, defining a unique mechanism of fidelity modulation. Likely a consequence of co-evolution of the NTD and the RdRP, the two parts of pestivirus NS5B with distinct origins have worked coordinately and created a unique mode of intra-molecular fidelity modulation.

## MATERIALS AND METHODS

### Plasmid construction and protein production

The DNA fragment corresponding to the NS5B residues 1–694 were amplified from the CSFV DNA clone pSM (Shimen strain) and cloned into a pET26b vector. The resulting plasmid pET26b-CSFV-NS5B was used as the template for construction of all mutant plasmids. NS5B point mutations were introduced by using the QuickChange site-directed mutagenesis method (45). N-terminal and C-terminal deletions were achieved through a site-directed ligase-independent mutagenesis (SLIM) method (46). All plasmids were transformed into *Escherichia coli* strain BL21-CondonPlus(DE3)-RIL for overexpression. Cells were grown overnight at 30 °C in the NZCYM medium with 25 μg/ml kanamycin (KAN25) and 17 μg/ml chloramphenicol (CHL17). The overnight culture was used to inoculate 1 l of NZCYM medium with KAN25 and CHL17. The cells were grown at 37 °C until the OD_600_ reached 0.6, and then were cooled to 25 °C. Isopropyl-β-d-thiogalactopyranoside (IPTG) was added at a final concentration of 0.5 mM, and the cells were grown for an additional 6 h before harvesting. Each NS5B construct contains a C-terminal hexahistidine tag.

### Purification of the CSFV NS5B and its variants

Cell lysis, protein purification and protein storage were performed as previously described for the JEV NS5 study (41), except that Tris (pH 7.0) was used as the buffering agent in the cation exchange chromatography and the final protein samples were stored in a buffer with higher concentration of NaCl (500 mM) and 10% (v/v) glycerol. The molar extinction coefficient for the NS5B constructs were calculated based on protein sequence using the ExPASy ProtParam program (http://www.expasy.ch/tools/protparam.html). The yield is typically about 15 mg of pure protein per liter of bacterial culture.

### Protein crystallization, diffraction data collection and structure determination

Crystals of the wild-type (WT) CSFV NS5B or its variant were grown by sitting drop vapor diffusion at 16 °C using 8 and 10 mg/ml protein. Within 2 weeks, quadrangular-shape crystals (form 1) grew with a precipitant solution containing 0.1 M Tris (pH 8.0) and 60% (v/v) poly(propylene glycol) 400 for WT, C-682 mutant and C-672 mutant, while spindle-shaped crystals (form 2) grew with a precipitant solution containing 0.1 M LiOAc, 0.1 M Bis–Tris (pH 6.0) and 20% (w/v) SOKLAN CP42 for C-672_AA mutant. The growth of form 1 crystals were further optimized by supplementing the precipitant solution with 10–30% volume of solution containing 0.1 M Tris (pH 8.0), 15% (w/v) polyvinylpyrrolidone, and 25% (w/v) poly(ethylene glycol) 5000 methyl ether for WT, 0.2 M NaCl, 0.1 M MES (pH 6.0), 45% (v/v) pentaerythritol propoxylate for C-682 mutant, or 0.2 M HEPES (pH 6.5), 10% (v/v) Jeffamine M-2005 for C-672 mutant. Crystals were flash cooled except that the C-672_AA crystals were transferred to a cryo-solution (precipitant solutions supplemented with 20% (v/v) glycerol) by incremental buffer exchange prior to flash cooling in liquid nitrogen.

Single crystal X-ray diffraction data were collected at the Shanghai Synchrotron Radiation Facility (SSRF) beamlines BL17U1 (wavelength = 0.9792 Å, temperature = 100 K) and BL19U1 (wavelength = 0.9785 Å, temperature = 100 K). At least 130–150° of data were typically collected in 0.3–0.5° oscillation steps. Reflections were integrated, merged and scaled using HKL2000 (Table 1) (47). The initial structure solution was obtained using the molecular replacement program PHASER (48) using coordinates derived from BVDV NS5B structures (PDB entries 1S4F and 2CJQ) as the search model (38,39). Manual model building and structure refinement were done using Coot and Phenix, respectively (49,50). The 3,500 K composite simulated-annealing omit 2*F*_o_ – *F*_c_ electron density maps were generated using CNS (51). Unless otherwise indicated, all polymerase superimpositions were done using the maximum likelihood based structure superpositioning program THESEUS (52).

**Table 1.** X-ray diffraction data collection and structure refinement statistics

PDB - construct - global conf.	5YF5 - WT - ‘closed’	5YF6 - C-682 - ‘closed’	5YF7 - C-672 - ‘closed’	5YF8 - C-672_AA - ‘open’
**Data collection** ^a^				
Space group	*P* 4_3_ 2_1_ 2	*P* 4_3_ 2_1_ 2	*P* 4_3_ 2_1_ 2	*I* 4_1_ 2 2
Cell dimensions				
*a, b, c* (Å)	160.0, 160.0, 55.2	161.4, 161.4, 56.0	162.2, 162.2, 56.8	115.8, 115.8, 393.9
α, β, γ (°)	90, 90, 90	90, 90, 90	90, 90, 90	90, 90, 90
Resolution (Å)^b^	60.0–2.50 (2.59–2.50)	50.0–2.10 (2.18–2.10)	50.0–2.27 (2.35–2.27)	60.0–3.40 (3.52–3.40)
No. unique reflections	25 700	43 794	35 366	18 873
*R*_merge_	0.080 (0.52)	0.066 (0.46)	0.075 (0.49)	0.128 (0.52)
*R*_meas_	0.087 (0.56)	0.070 (0.48)	0.078 (0.51)	0.142 (0.58)
*I* / σ*I*	22.0 (4.1)	34.3 (6.0)	34.3 (4.5)	11.7 (3.2)
Completeness (%)	99.7 (100.0)	100.0 (100.0)	99.2 (98.3)	98.8 (99.5)
Redundancy	6.7 (6.8)	10.3 (10.3)	12.7 (12.4)	5.3 (5.1)
**Structure refinement**				
Resolution (Å)	2.50	2.10	2.27	3.40
No. unique reflections	25 631	43 679	35 259	18 708
*R*_work_ / *R*_free_^c^ (%)	18.5 / 24.2	18.0 / 22.7	19.9 / 24.5	23.0 / 27.1
No. atoms				
Protein	5 016	5 108	5 124	4 839
Ligand/Ion/Water	/ / 81	/ / 344	/ / 211	28 / / 9
*B*-factors (Å^2^)				
Protein	52.4	40.2	52.4	71.3
Ligand/Ion/water	/ / 45.4	/ / 41.6	/ / 47.6	69.5 / / 63.4
R.m.s. deviations				
Bond lengths (Å)	0.008	0.007	0.007	0.010
Bond angles (°)	0.918	0.800	0.826	1.164
Ramachandran stat.^d^	91.1 / 8.5 / 0.2 / 0.2	91.9 / 7.9 / 0.2 / 0.0	91.1 / 8.6 / 0.3 / 0.0	82.6 / 16.1 / 0.9 / 0.4

^a^One crystal was used for data collection for each structure.

^b^Values in parentheses are for the highest-resolution shell.

^c^5% of data are taken for the R_free_ set, and the same R_free_ set is applied for the WT, C-682 and C-672 structures.

^d^Values are in percentage and are for most favored, additionally allowed, generously allowed, and disallowed regions in Ramachandran plots, respectively.

### RNA preparation

The chemically synthesized 30-mer template strand (T30, Integrated DNA Technologies) was purified by 12% (w/v) polyacrylamide/7 M urea gel electrophoresis, excised from the gels, and electro-eluted by an Elu-Trap device (GE Healthcare). Purified T30 was stored in an RNA annealing buffer (RAB: 50 mM NaCl, 5 mM Tris (pH 7.5), 5 mM MgCl_2_) at −80 °C after a self annealing process (a 3-min incubation at 95 °C followed by snap-cooling to minimize intermolecular annealing). For all the *in vitro* RdRP assays, T30 was annealed with a GG dinucleotide primer bearing a 5′-phosphate (P2, Jena BioSciences) at a 1:1.25 molar ratio via a 3-min incubation at 45 °C followed by slow-cooling to r.t. in the RAB to yield the T30/P2 construct.

### The dinucleotide-driven RdRP assays

A regular 20-μl reaction mixture containing 4 μM T30/P2 construct (including 5 μM P2), 15 μM extra P2, 6 μM CSFV NS5B, 300 μM ATP, 300 μM UTP, 20 mM NaCl, 50 mM Tris (pH 7.0), 5 mM MgCl_2_, 5 mM dithiothreitol (DTT), was incubated at 30 °C for 45 min. Reaction quenching, sample processing, denaturing polyacrylamide gel electrophoresis (PAGE), RNA visualization by Stains-All (Sigma-Aldrich) staining and quantification were as previously described in a JEV RdRP study (53). All Stains-All based gels were shown in greyscale-mode by converting from the original RGB-mode without any brightness/contrast adjustment.

Two types of misincorporation assays were carried out derived from the regular assays described above, corresponding to a guanosine-directed UMP misincorporation (G:U_mis_) at the 10th nucleotide of the product or a cytosine-directed UMP misincorporation (C:U_mis_) at the 11th nucleotide of the product. For radioactive labeling, [α-^32^P]ATP (PerkinElmer Life Sciences) was supplied with ATP/UTP (300 μM each) for the G:U_mis_ assays and with ATP/UTP/CTP (300 μM each) for the C:U_mis_ assays. For the G:U_mis_ and C:U_mis_ assays, experiments were either performed in a time course format (typically seven time points) for representative NS5B constructs or with two representative time points for all constructs. The snG:U_mis_ assays were performed in a two-step format. In the first step, the reaction proceeded for 45 min in the presence of ATP/UTP (300 μM each), the mixture was then centrifuged at 14 549 g and the supernatant was removed, and the precipitate containing the NS5B elongation complex (EC) was washed twice with a reaction buffer (20 mM NaCl, 50 mM Tris (pH 7.0), 5 mM MgCl_2_, 5 mM DTT). In the second step, the UMP misincorporation reaction was conducted at 30 °C at various UTP concentrations in the range between 50 and 1600 μM. Reaction was quenched at various time points. The radioactive RNA products in the G:U_mis_ and C:U_mis_ assays were visualized by a Cyclone Plus Storage Phosphor System (PerminElmer Life Sciences) and regular products in the snG:U_mis_ assays were visualized by Stains-All staining. Band intensity quantification was performed using ImageJ (https://imagej.nih.gov/ij). To estimate the single-nucleotide misincorporation rate (*r*_mis_) corresponding to the conversion from 9-mer to 10-mer in the snG:U_mis_ assays, the values representing the fraction of 10-mer intensity (*f*) at all time points (*t*) was fitted to a single exponential rise equation: *f* = offset + amplitude [1 – exp(–*r*_mis_ × *t*)], where offset is related the portion of 10-mer contributed by minimum amount of G:U_mis_ prior to the addition of UTP and the amplitude is related to the possibility of a 9-mer that eventually failed to extend to an 10-mer. The *r*_mis_ values obtained under different UTP concentrations ([S]) were fitted to the Michaelis–Menten type equation: *r_mis_* = *k*_mis_ × [S]/(*K*_M_^app^ + [S]), where *k*_mis_ is the G:U_mis_ rate constant and *K*_M_^app^ is the Michaelis constant for UTP.

The snG:C assays assessing the P9 to P10 conversion rate and the stability assays were performed in a two-step format as in the snG:U assays and with the first step identical to the snG:U_mis_ assays. In the second step of the snG:C assays, the precipitate was resuspended with the regular reaction buffer or reaction buffer with NaCl concentration elevated to 200 mM. The snG:C incorporation was quenched immediate (0 min) following the manual mixing of CTP (for a final concentration of 300 μM) or after 1 min. In the second step of the stability assays, the precipitate was resuspended with a high salt buffer (200/500 mM NaCl, 50 mM Tris (pH 7.0), 5 mM MgCl_2_, 5 mM DTT), and incubated at 37 °C for 0 to 7 days. Following the incubation, CTP was supplied at 300 μM final concentration and the reaction proceeded for 1 min at 30 °C. After subtracting the intensity of the P10 misincorporation product from the first step, the intensity fraction of P10 among the total amount of P9 and P10 ([P10_int_ – P10_m,int_]/[P9_int_ + P10_int_ – P10_m,int_]) was used to estimate the fraction of the NS5B EC survived the incubation.

## RESULTS

### An anatomy of the CSFV NS5B

**Figure 1.**
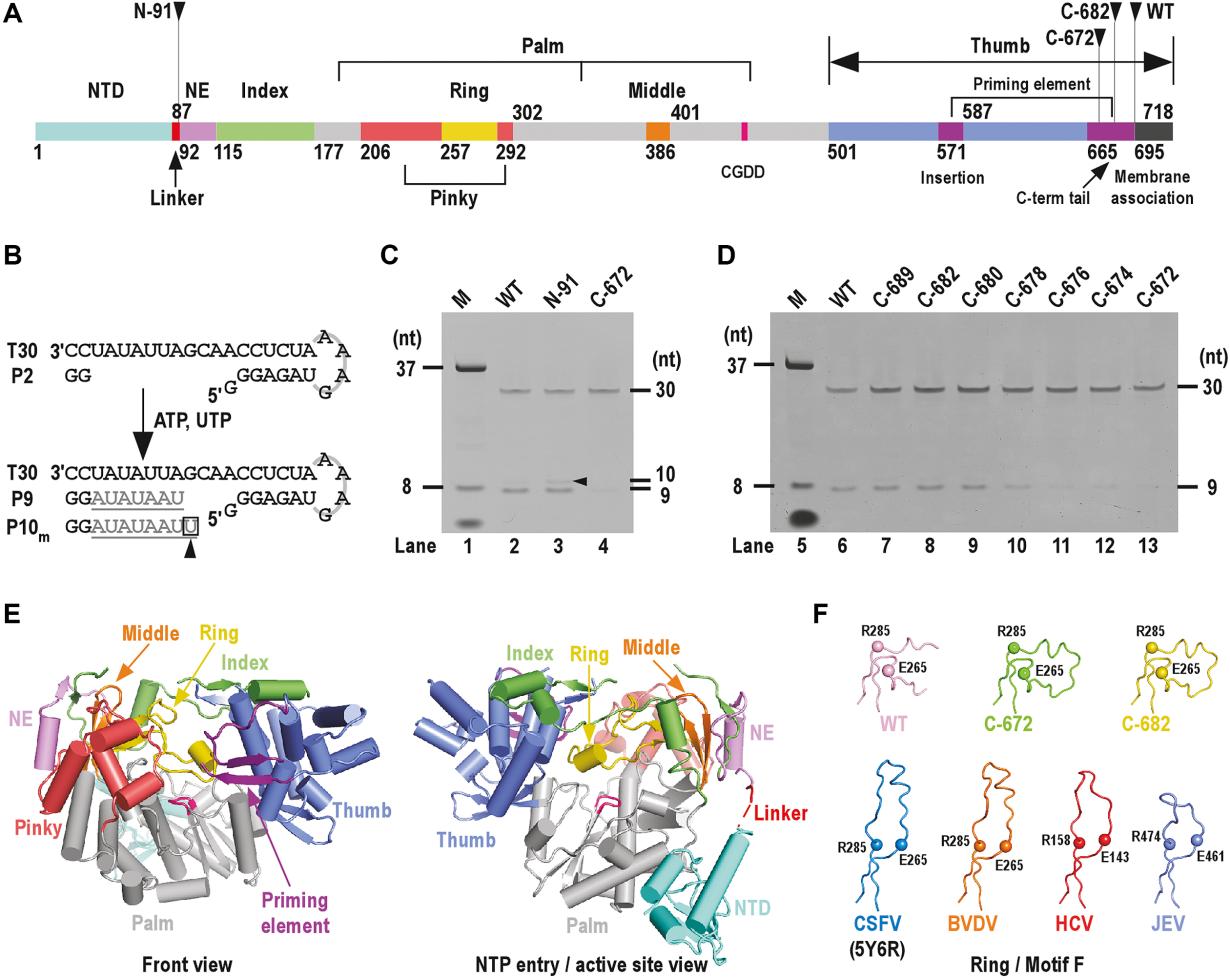
Functional/structural element assignment, the *de novo* RdRP activity assessment and the global structure of CSFV NS5B. ( **A**) A color-coded bar of functional or structural elements of CSFV NS5B. Coloring scheme: NTD in teal, linker in red, RdRP palm in grey, N-terminal extension (NE) in violet, index finger in green, middle finger in orange, ring finger in yellow, pinky finger in light red, thumb in slate, priming element in purple, motif C signature sequence CGDD in magenta and membrane association region in black. The numbers defining the residue ranges of each element are shown. (**B**) A schematic diagram of the *de novo*-mode RdRP assays. Construct T30/P2 was used as the RNA substrate. When ATP and UTP were supplied as the only NTP substrates, the template-strand T30 directed a 7-nucleotide (grey) extension of the dinucleotide primer P2 (black) to produce a 9-mer product (P9). The 10-mer product was generated through a G:U_mis_ event. (**C** and **D**) Comparison of the *de novo* RdRP activity for the WT NS5B and its N-/C-terminal truncated forms. The oval-shaped band below the 8-nt marker (M) is the bromphenol blue mixed with the marker sample. Note that the 8-nt marker was chemically synthesized and bearing hydroxyl groups at the 5′-end, and therefore migrated slower than the 9-nt product bearing a 5′-phosphate. (**E**) Global views of CSFV NS5B crystal structure. Structure of NS5B C-682 construct shown in orientations viewing into the front channel (left) and NTP entry channel (right). The coloring scheme is consistent with that in panel-A. (**F**) A structural comparison of the *Flaviviridae* RdRP ring finger (motif F). The ring finger is shown as noodles with the α-carbon atoms of two highly conserved motif F residues in spheres. Top row: CSFV NS5B constructs; bottom row: representative *Flaviviridae* NS5B constructs. PDB entries: 5YF5 (WT); 5YF6 (C-682); 5YF7 (C-672); 5Y6R (CSFV) (42); 1S4F (BVDV) (39); 1NB4 (HCV) (59); 4K6M (JEV) (41).

Crystal structures of the highly homologous BVDV NS5B (sequence identity = 71%) and related flavivirus NS5 help designate residues 115–694 as the RdRP core of the 718-residue CSFV NS5B (Figure 1A) (39,41,54). For better description of the RdRP structure, we followed a nomenclature first used for describing the picornavirus RdRPs by defining individual finger subdomains as index, middle, ring and pinky (Figure 1A) (12). Similar to HCV NS5B but different from flavivirus NS5, the pestivirus RdRP thumb domain contains a two-component priming element: an insertion (residues 571–586) between two thumb helices and a C-terminal tail (residues 665–694) (Figure 1A). This priming element of the *Flaviviridae* RdRP plays essential roles in *de novo* initiation through interactions with the template RNA and the initiating NTPs (26,55,56). Residues beyond the RdRP core can be divided into three regions. Residues 1–91 containing the NTD are not included in the BVDV NS5B constructs used to determine the crystal structures (38,39). Residues 92–114 are structurally analogous to the ‘N-terminal extension’ (NE) of the flavivirus NS5 that contributes to the flavivirus RdRP activity (39,41,53). Residues 695–718 at the C-terminus are highly hydrophobic and are likely analogous to the membrane anchoring helix of the HCV NS5B (44,57).

### The NTD of CSFV NS5B is not required for *de novo* RNA synthesis but may act as a fidelity modulator

In order to investigate the function of the NTD of CSFV NS5B, we first made an NS5B construct comprising residues 1–694 with only the C-terminal hydrophobic region removed. For description purpose, we herein named this construct as the wild-type (WT). This construct was soluble and capable in GG dinucleotide driven RNA synthesis using a template RNA sequence derived from the RdRP assays established in the HCV NS5B and the JEV NS5 (53,58) (Figure 1B and C). This type of assays, although not identical to the *de novo* initiation assays, have been typically used to assess *de novo* mode RNA synthesis by viral RdRPs and are different from the assays using longer oligonucleotides as primers. Using a 30-mer RNA template (T30), we compared the RdRP activity both in the presence and absence of the dinucleotide primer (P2), and found that in the latter case the overall activity was low and sequences of the dominant products were not faithfully directed by the template sequence (Supplementary Figure S1). Hence, we decided to use the P2-based assays as primary approaches for the *in vitro* characterization of NS5B. When T30 and P2 were used to generate the T30/P2 RNA substrate, a 9-mer product (P9) was expected when ATP and UTP were provided as the only NTP substrates (Figure 1B). Deletion of the NTD (Figure 1A, construct N-91) from the WT backbone did not apparently affect the P9 product level (Figure 1C, compare lanes 2 and 3). In contrast, deletion of residue 673–694 (Figure 1A, construct C-672) in the C-terminal tail resulted in apparent reduction of product level (Figure 1C, compare lanes 2 and 4). In order to precisely determine which region within residues 673–694 is critical for *de novo* synthesis, incremental C-terminal truncations (Figure 1D, seven constructs from C-674 to C-689) were made on the WT backbone. The results indicated that residues 681–694 are not essential, as residue removal in this region did not apparently affect product level (Figure 1D, compare lanes 7–9 to lane 6). Further truncations beyond residue 681 led to apparent reduction of product level (Figure 1D, compare lanes 10–13 to lane 6), suggesting that the N-terminal half (residues 665–680) of the C-terminal tail is likely required for optimal *de novo* synthesis.

Very interestingly, the removal of NTD resulted in obvious higher level of a 10-mer misincorporation product (P10_m_) (Figure 1C, compare lanes 2 and 3), indicating that it likely plays important roles in controlling RdRP fidelity. In the following sections, crystallography and enzymology were utilized to dissect the structure and function of NTD. All NS5B constructs used in following polymerase assays have intact C-terminal tail (i.e. ending at residue 694) to preserve the capability of *de novo*–mode synthesis.

### The structure of the CSFV RdRP core is largely consistent with the BVDV structures

With an aim to study the structure-function relationship of the CSFV NS5B including the NTD, we screened crystallization conditions of three constructs (WT, C-682, C-672) and obtained single crystals for all constructs after multiple rounds of optimization from a single initial crystallization condition. The structure of the WT was solved at 2.5 Å resolution in space group *P*4_3_2_1_2 by molecular replacement using a BVDV NS5B structure comprising residues 91–672 as the search model (39) (Table 1). The structures of the C-682 and C-672 were solved at 2.1 and 2.3 Å resolution, respectively, in the same crystal form by molecular replacement using the structure of the WT as the search model (Table 1). These three structures, each containing one NS5B molecule in the crystallographic asymmetric unit, are highly similar with root mean square deviation (RMSD) values of 0.4–0.6 Å between the WT and two C-terminal truncation mutants for all superimposable α-carbon atoms with 99% coverage of the resolved residues in the structure of the WT. We hereinafter choose the highest-resolution C-682 structure as the primary structure for illustration with difference between structures discussed where necessary (Figure 1E and F). In the structures of the WT and C-682, residues beyond 672 are disordered. Therefore, all three structures are not sufficient to provide a structural basis for why residues up to residue 680 are essential for the *de novo*-mode synthesis, but are valid for assessing the NTD–RdRP interactions described as follows.

The CSFV NS5B structures are relatively complete, with more than 640 residues resolved for all 672–694 NS5B residues (Figure 1E). The disordered regions mainly include residues 1–12 at the N-terminus, residues 127–130 in the index finger, residues 532–537 in the thumb and C-terminal residues beyond position 671. For some of the constructs, the tips of the ring finger (residues 257–291) and the loop-like priming element insertion (residues 571–586) are disordered. The RdRP core of CSFV NS5B is structurally consistent with the BVDV structures, with an RMSD value of 1.5 Å (92% coverage) between the BVDV NS5B N438 duplication mutant structure and the CSFV C-682 structure (39). Aside from global structural difference brought by small-scale rigid body movement between the RdRP domains, the most notable difference is the conformation of the ring finger. Different from the observations in the WT BVDV (38), full-length JEV (41), HCV (59) and the recently reported CSFV RdRP structures (42) that have a canonical fold optimal for NTP entry and binding, the CSFV NS5B ring finger bent toward the pinky finger, partially occupying the template RNA binding channel (Figure 1F). Although this conformation is likely not compatible with *de novo* initiation, normal activity of the WT and C-682 observed in the polymerase assays suggests that the canonical conformation also exist in solution and may be in equilibrium with the observed bent conformation observed in the crystal structures (Figure 1C and D).

### The NTD folds into a unique globular domain and interacts with the RdRP palm

**Figure 2.**
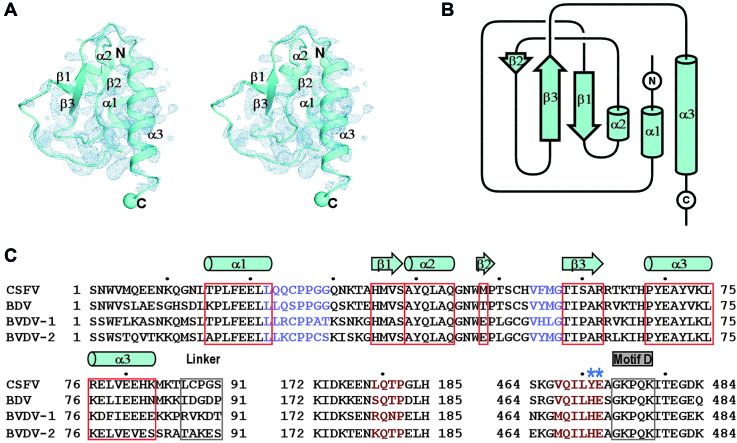
The structure, folding topology and sequence analysis of the CSFV NS5B NTD. (**A**) Stereo-pair images of the NTD with 3500 K composite SA-omit electron density map (contoured at 1.2σ) overlaid. (**B**) Topology of the CSFV NS5B NTD. The α-helix and β-strand labeling in panels A and B is based on secondary structure assignment by DSSP (68). (**C**) The sequence alignment of the pestiviruses NS5B NTD, linker and the RdRP regions that interact with NTD. The symbols of NTD α-helices (cylinders) and β-strands (block arrows) are plotted based on the secondary structure assignment of the CSFV NS5B structure. Two key residues involved in the intra-molecular of NTD–RdRP interactions and chosen for mutagenesis are indicated by asterisks. UniProtKB accession numbers of the pestivirus NS5B sequences used: Q5U8X5 (CSFV), X2KMP2 (BDV), P19711 (BVDV-1) and A0A0M4S9G5 (BVDV-2).

**Figure 3.**
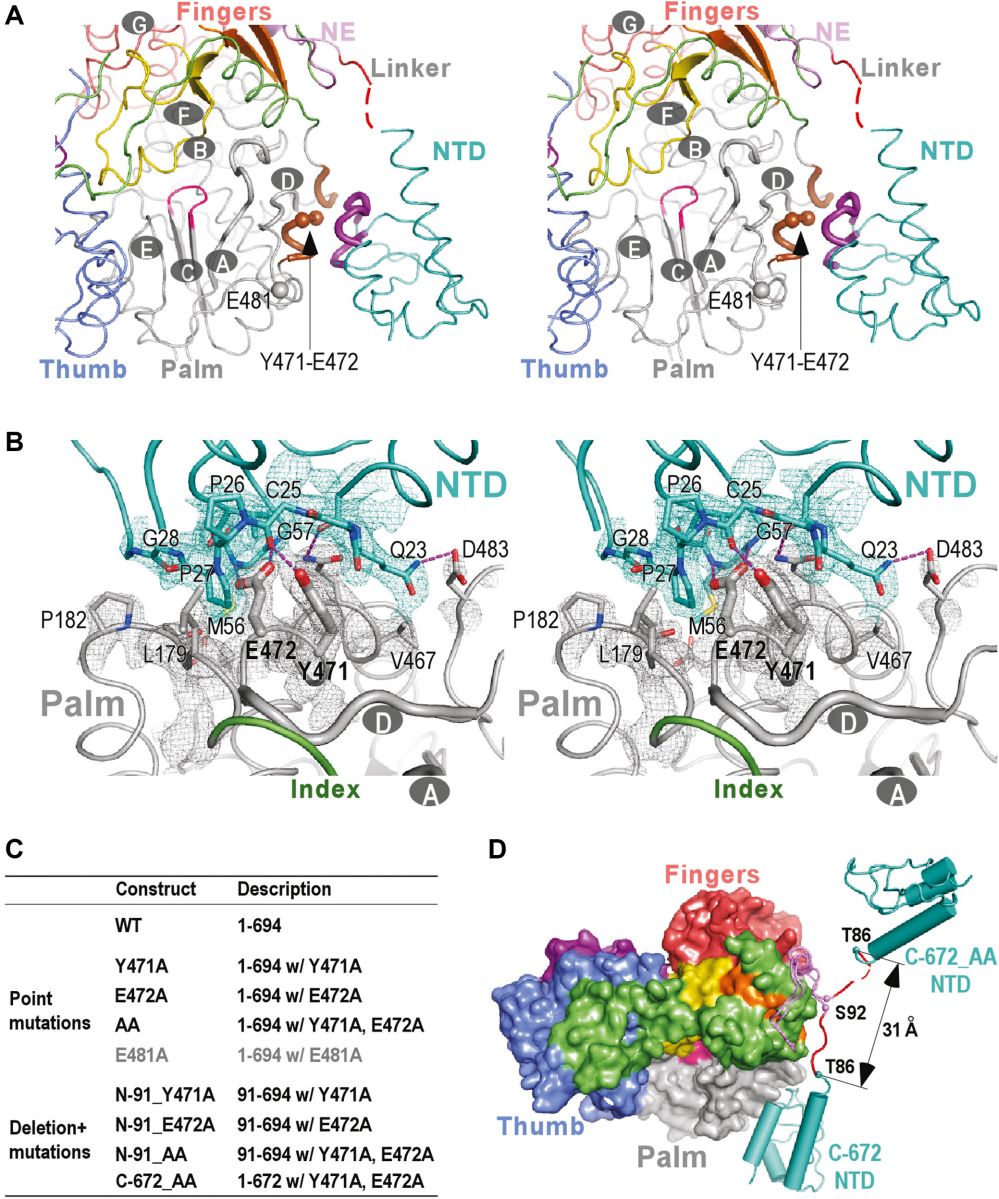
The intra-molecular NTD–RdRP interface and an open-conformation NS5B structure derived from the interface mutations. (**A**) Stereo-pair images of CSFV NS5B structures viewing from the NTP entry channel. The seven CSFV RdRP catalytic motifs A-G are labeled in the structures, and motifs A and D are shown as thick noodles. The key regions from NTD and RdRP that form the NTD–RdRP interface are colored in purple and brown, respectively, while all other regions are colored as in Figure 1A. The α-carbon atoms of residues 471, 472 and 481 are shown as spheres. Red dotted line in the structure indicates residues 87–88 that were not modeled due to poorly defined electron density. (**B**) Stereo-pair images of the NTD–RdRP interface with 3500 K composite SA-omit electron density map (contoured at 1.5σ) in the vicinity of the interface overlaid. Side chains of residues 471–472 (thick) and other residues (thin) that participate in the interface interactions are shown in sticks. The hydrogen bonds are shown as purple dotted lines. (**C**) A list of NS5B constructs used to investigate the structure and function of NTD, with full descriptions including residue range, mutation site and mutation type. (**D**) A comparison of the global conformations observed in the crystal structures of the CSFV NS5B C-672 and the interface mutant C-672_AA. NTD and linker are shown in noodle/cylinder style, and RdRP is shown as surface representation. The α-carbon atoms of residues T86 and S92 adjacent to the NTD–RdRP linker are shown as spheres. The coloring scheme is as in Figure 1A.

The global conformation of our CSFV NS5B structures is consistent with the recently reported CSFV structure with an RMSD value of 0.9 Å (95% coverage) between the reported structure and our representative C-682 structure (42). These structures together reveal that the NTD adopts a globular α/β fold with an α-β-α-β-β-α pattern (Figure 2). We were not able to identify any known structural domain highly homologous to NTD using the DALI server (60). Taking the consideration that sequence homology of NTD also has not been reported beyond pestiviruses, NTD therefore represents a highly unique viral RdRP fusion partner that may play important regulatory roles to the RdRP function. The NTD interacts with the palm of the RdRP core intra-molecularly with a mixture of hydrophobic and hydrogen bonding interactions (Figure 3A and B). These interactions mainly involve NTD residues 22–29 and 54–57 and the RdRP core residues 179–182 and 467–472 in the vicinity of motifs A and D, occluding ∼1280 Å^2^ of solvent accessible surface area (Figure 3B). Lying in the heart of this NTD–RdRP interface, are two adjacent RdRP residues Y471 and E472. The aromatic ring of Y471 side chain is wrapped around by NTD resides 23–27 through hydrophobic interactions and its phenyl hydroxyl group forms a hydrogen bond with the carbonyl oxygen of C25 backbone, while the carboxyl group of the E472 side chain forms two hydrogen bonds with backbone amide nitrogen atoms of residues M56 and G57 and its two-carbon aliphatic side chain region interacts with P27 and V57 through hydrophobic interactions (Figure 3B). The intra-molecular NTD interactions with the RdRP palm that controls the active site closure, together with the observation of the NTD deletion mutant N-91 exhibited higher level of misincorporation in the *de novo* RNA synthesis, suggesting a unique mode of fidelity modulation. The NTD–RdRP interface is different from the two types of the methyltransferase (MTase)-RdRP interface first identified in the NS5 of JEV and DENV serotype 3 (DENV3) that occlude relatively large surface areas (1500–1600 Å^2^) (41,61). Firstly, the flavirus MTase interacts with the RdRP fingers domain, while the pestivirus NTD interacts with the RdRP palm. Secondly, the nature of the interface interactions is different, with the JEV-interface featuring a conserved hydrophobic core, the DENV3-interface being primarily polar, and the CSFV-interface having a mixture of interactions as mentioned above.

### Some of the Mutations designed to perturb the NTD–RdRP intra-molecular interface led to crystallization of NS5B in an ‘open’ conformation with the interface fully disrupted

To better understand the nature of the NTD–RdRP intra-molecular interface interactions and whether they regulate the RdRP fidelity, we designed point mutations at the Y471 and E472 sites in the context of the WT NS5B and the N- and C-terminal deletion mutants (Figure 3C). Crystallization screenings were performed for all the NTD containing mutant constructs and four of them were successfully crystallized with one construct crystallized under two different conditions (Table 1 and Supplementary Table S1). Among these five structures, three of them (Y471A and two forms of C-672_Y471A) maintain the WT conformation with the NTD–RdRP intra-molecular interface maintained. The other two structures, obtained using the C-672 construct bearing the Y471A-E472A double mutation (C-672_AA) and the C-672 construct with the E472A single mutation (C-672_E472A), were solved in a space group different from those of the WT, C-682 and C-672 structures (Table 1 and Supplementary Table S1) (Figure 3D). Very interestingly, the C-672_AA and C-672_E472A adopt a drastically different global conformation with the NTD–RdRP interface no longer maintained (C-672_AA structure shown in Figure 3D). The NTD–RdRP intra-molecular interactions are fully disrupted, and the NTD is rather involved in a non-intensive three-way interaction involving two symmetry-related neighboring NS5B molecules in the crystal lattice. These structural data together suggest that the selection of Y471-E472 mutation sites is valid in perturbing the NTD–RdRP interface, but the interface may not necessarily be fully disrupted by some of the mutations.

### Perturbing the NTD–RdRP intra-molecular interactions reduced the fidelity of CSFV NS5B

**Figure 4.**
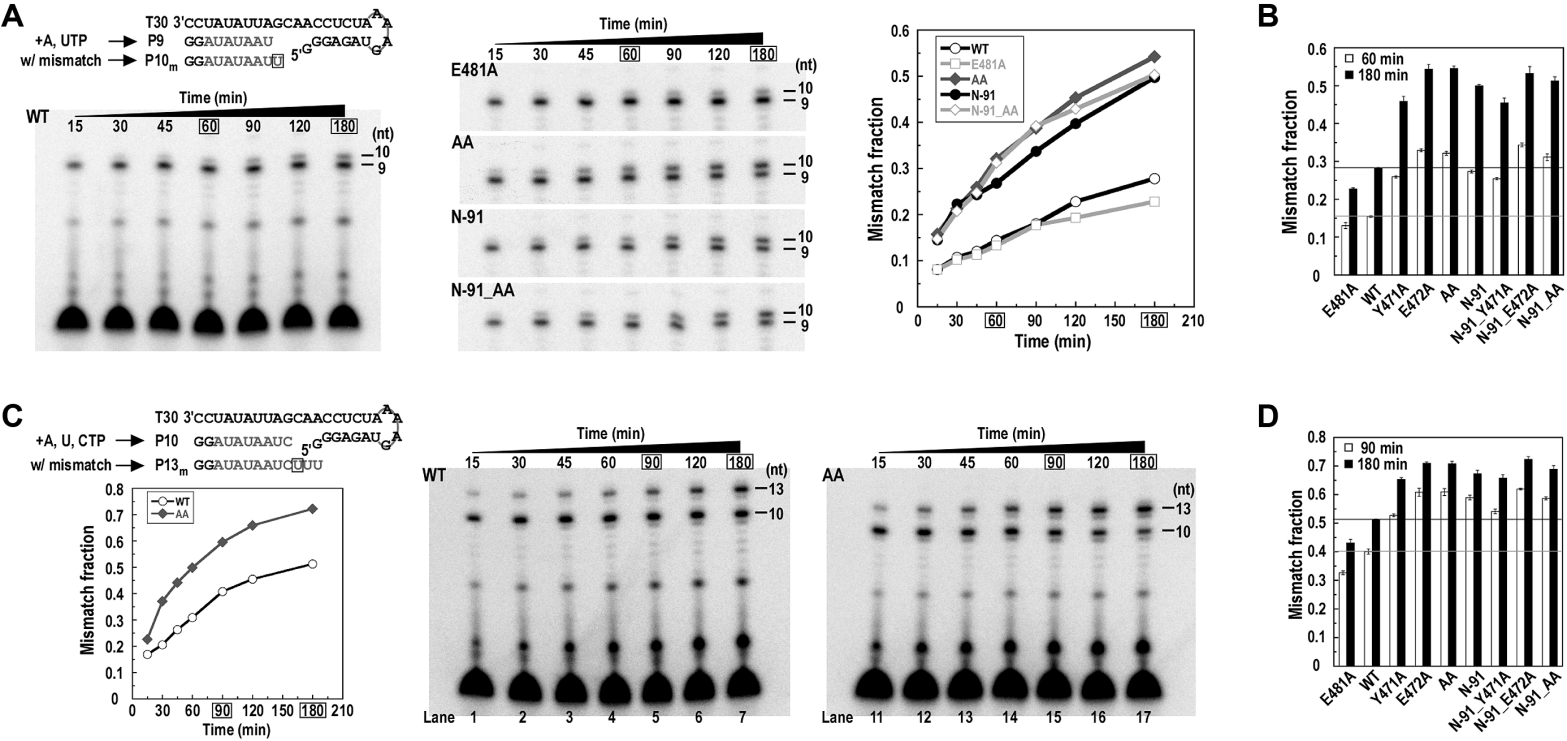
NS5B fidelity characterization using the G:U_mis_ and C:U_mis_ assays. (**A**) Monitoring the G:U_mis_ in a time course manner for five representative NS5B constructs. The mismatch fraction ([P10_m_]/([P9]+[P10_m_])) values were calculated to generate the line plot, where P10_m_ is the G:U_mis_ derived 10-mer product and P9 is the regular 9-mer product under the experimental setting. (**B**) Monitoring the G:U_mis_ at two representative reaction time points. Each data series was taken from three individual experimental sets. Average mismatch fraction and standard deviation are shown in the column chart. The two time points (60 min and 180 min) in the panel-A experiments chosen for tests in panel B were circled by rectangles. (**C**) Monitoring the C:U_mis_ in a time course manner for two representative NS5B constructs. The mismatch fraction ([P11_m_-P13_m_]/([P10–13_m_])) values were calculated to generate the line plot, where P10 is the regular product and P11_m_ – P13_m_ are the C:U_mis_ derived 11–13-mer products under the experimental setting. (**D**) Monitoring the C:U_mis_ at two representative reaction time points. Each data series was taken from three individual experimental sets. Average mismatch fraction, standard deviation and gels from one set are shown in the column chart. The two time points (90 and 180 min) in the panel C experiments chosen for tests in panel D were circled by rectangles. For the column charts in panels B and D, one set of representative gels are provided in the Supplementary Figure S3, A and B, respectively.

In order to quantitatively assess whether the fidelity levels are modulated by the intra-molecular NTD–RdRP interface interactions, we established the ^32^P-radioactivity based NTP misincorporation assays using the T30/P2 construct utilized in the aforementioned *de novo*-mode synthesis assessment (Figure 1B and C). When ATP and UTP were provided as the only NTP substrates, the 9-mer product (P9) was expected based on correct NMP incorporation. The slow accumulation of the 10-mer product (P10_m_) was derived from a G:U_mis_ event (see Material and Methods), since providing UTP but not ATP as the only substrate to the P9-containing complex led to slow accumulation of 10-mer (Supplementary Figure S2). We therefore used molar fraction of G:U_mis_-derived P10_m_ among the total amount of P9 and P10_m_ (mismatch fraction) to assess the fidelity of NS5B (Figure 4A). In a time course reaction, WT NS5B exhibited low level of misincorporation with the mismatch fraction gradually increasing over time and reached about 0.25 at the 180-min time point. When the NTD was absent (constructs N-91 and N-91_AA) or alanine mutations were simultaneously introduced at residues 471 and 472 (construct AA), NS5B consistently exhibited higher level of misincorporation than the WT did, with mismatch fractions around 0.5 at the 180-min time point. When an alanine mutation was introduced at residue E481 that is also on the RdRP surface but does not participate in the NTD–RdRP intra-molecular interactions (Figure 3A and C), the misincorporation level is comparable to that of the WT (Figure 4A). 60-min and 180-min were chosen as representative time points for a comparison also including single-point alanine mutants (Y471A, E472A, and the N-91 form mutants) (Figure 4B and Supplementary Figure S3A). Consistent with the observation in the time course experiments, every mutant either lacking the NTD or bearing mutation(s) at residues Y471 and E472 showed much higher level of misincorporation than the WT did. The effect of E472A mutation was highly consistent with AA mutation (Figure 4B and Supplementary Figure S3A, compare lanes 4/8/14/18 to 5/9/15/19), while the effect of Y471A mutation was slightly smaller than that of the E472A and AA mutations (Figure 4B and Supplementary Figure S3A, compare lanes 3/7/13/17 to 4/8/14/18 and 5/9/15/19).

Since polymerase misincorporation level can be affected by the type of misincorporation and the sequence context of the misincorporation site, we established the second type of regular NTP misincorporation assays using the same T30/P2 construct for a more adequate assessment of the RdRP fidelity modulation brought by the NTD. When ATP, UTP and CTP were provided as the only NTP substrates, NS5B was expected to synthesize a 10-mer product (P10) through correct NMP incorporation. A 13-mer was also observed primarily derived from a C:U_mis_ (See Material and Methods) followed by two correct UMP incorporation events, while only a minority of the 13-mer may arise from a cytosine-directed AMP misincorporation (C:A_mis_) or cytosine-directed CMP misincorporation (C:C_mis_) (Figure 4C and Supplementary Figure S2C and D). For simplicity, here we use C:U_mis_, the major misincorporation event, to describe these misincorporation assays. Similar to the observation in the G:U_mis_ assays, the AA mutant exhibited much higher misincorporation level than the WT did in the time course experiments (Figure 4C). At the 180-min time point, the AA mutant had a mismatch fraction ∼0.7 (defined by the molar fraction of 11–13-mer misincorporation products among the 10–13-mer products) while the WT had a value about 0.5 (Figure 4C and Supplementary Figure S3B, compare lanes 7 and 17). For assessment of all mutants, the 90-min and 180-min time points were chosen. The effect brought by NTD removal or point mutation(s) were consistent with that observed in the G:U_mis_ assays (Figure 4D and Supplementary Figure S3B). Overall, the mismatch fractions of C:U_mis_ reactions were higher than those of the G:U_mis_ reactions, likely reflecting the differences in misincorporation types (G:U versus C:U) and sequence contexts in these assays. Collectively, these biochemical data indicated that the RNA synthesis fidelity of CSFV NS5B is fine-tuned by its NTD through the intra-molecular interactions with the RdRP palm.

### Perturbing the NTD–RdRP intra-molecular interactions does not affect polymerase elongation complex (EC) processivity

**Figure 5.**
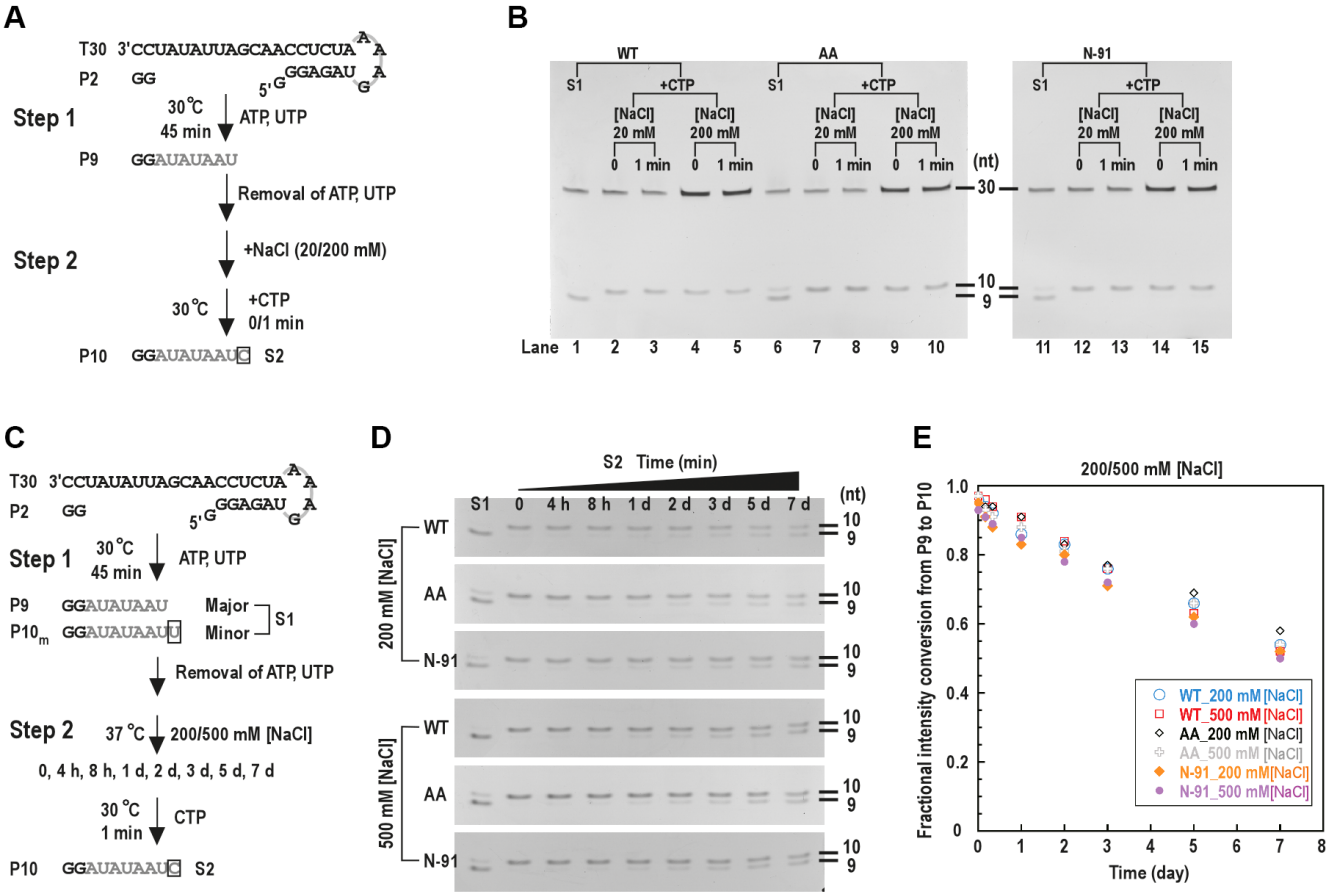
The processivity of NS5B EC was not affected by the NTD–RdRP interface mutations. (**A**) The reaction flow chart for the snG:C assays that revealed fast catalytic rate of P9 to P10 conversion. (**B**) In the snG:C assays, the P9 produced by NS5B constructs (WT, AA and N-91) in the first step rapidly extended to the P10 in the presence of the cognate nucleotide CTP under regular (20 mM) or high (200 mM) NaCl concentrations. (**C**) Reaction flow chart of the stability assays. (**D** and **E**) The P9-containing complexes formed by different NS5B constructs were incubated under high NaCl concentrations (200/500 mM) for different time period (up to 7 days) before the fraction of the complex survived the incubation was estimated by the fractional intensity conversion from P9 to P10 ([P10_int_ – P10_m,int_]/[P9_int_ + P10_int_ – P10_m,int_], see details in Materials and Methods) plotted in panel E.

The misincorporation events in both the G:U_mis_ and C:U_mis_ assays were coupled to the slow accumulation of the correct product (P9 and P10 in G:U_mis_ and C:U_mis_ assays, respectively) through P2-driven initiation. In order to assess the misincorporation solely occurred in the elongation phase and to find out whether processivity, another key polymerase property, was affected by the NTD–RdRP interface mutations, we first needed to explicitly assess whether the P9-containg complex has entered the elongation phase. As fast catalytic rate and high stability are the two hallmarks of a polymerase elongation complex (EC), we tested P9 to P10 single nucleotide extension (corresponding to the guanosine-directed CMP incorporation, or G:C) rate and the P9-containing complex stability of the WT NS5B (Figure 5). The single-nucleotide G:C (snG:C) assays was designed in a two-step format. In the first step ATP and UTP were supplied to generate the P9 containing complex, and CMP incorporation was initiated after the removal of the originally supplied ATP/UTP (Figure 5A). Immediately after the manual addition of CTP (corresponding to ‘0 min’), P9 to P10 conversion was complete, under two different NaCl concentrations tested (Figure 5B, lanes 2 and 4), suggesting that the catalytic rate of this single nucleotide addition is much faster than the P9 and P10 accumulation observed in the G:U_mis_ and C:U_mis_ assays. To test the stability of the P9-containing complex, we used NaCl and/or heparin as the challenging agent in the stability assays. When NaCl concentration was at least 100 mM, the P2-driven P9 formation with ATP/UTP was not detected (Supplementary Figure S4A and B). In contrast, the majority of the P9-containing complex survived long-time NaCl challenge at 200 mM or 500 mM concentration in the stability assays (Figure 5C–E). After a 1-day NaCl challenge, ∼85% of the P9 can be rapidly converted to P10 when CTP was supplied, while after a 7-day challenge still ∼54% of the P9 can be converted. When 100 μg/ml heparin or 100 μg/ml combined with 500 mM NaCl was supplied for the challenge, similar results were obtained (Supplementary Figure S4C–E). These data together suggest that the P9-containing complex is highly stable and can rapidly elongate and therefore can be considered as an EC.

In order to test whether perturbing the NTD–RdRP interface interactions affects the processivity of the EC, we carried out the snG:C assays and the stability assays for the double mutant AA and the NTD-truncated construct N-91. The results showed that both NS5B variants behaved similarly to WT, with very fast conversion of P9 to P10 (Figure 5B) and comparable stability upon challenge of NaCl and/or heparin (Figure 5D and E; Supplementary Figure S4D and E). These data indicate that EC processivity is likely not modulated by the NTD–RdRP intra-molecular interactions.

### The NTD regulates RdRP EC fidelity through catalysis but not NTP binding

**Figure 6.**
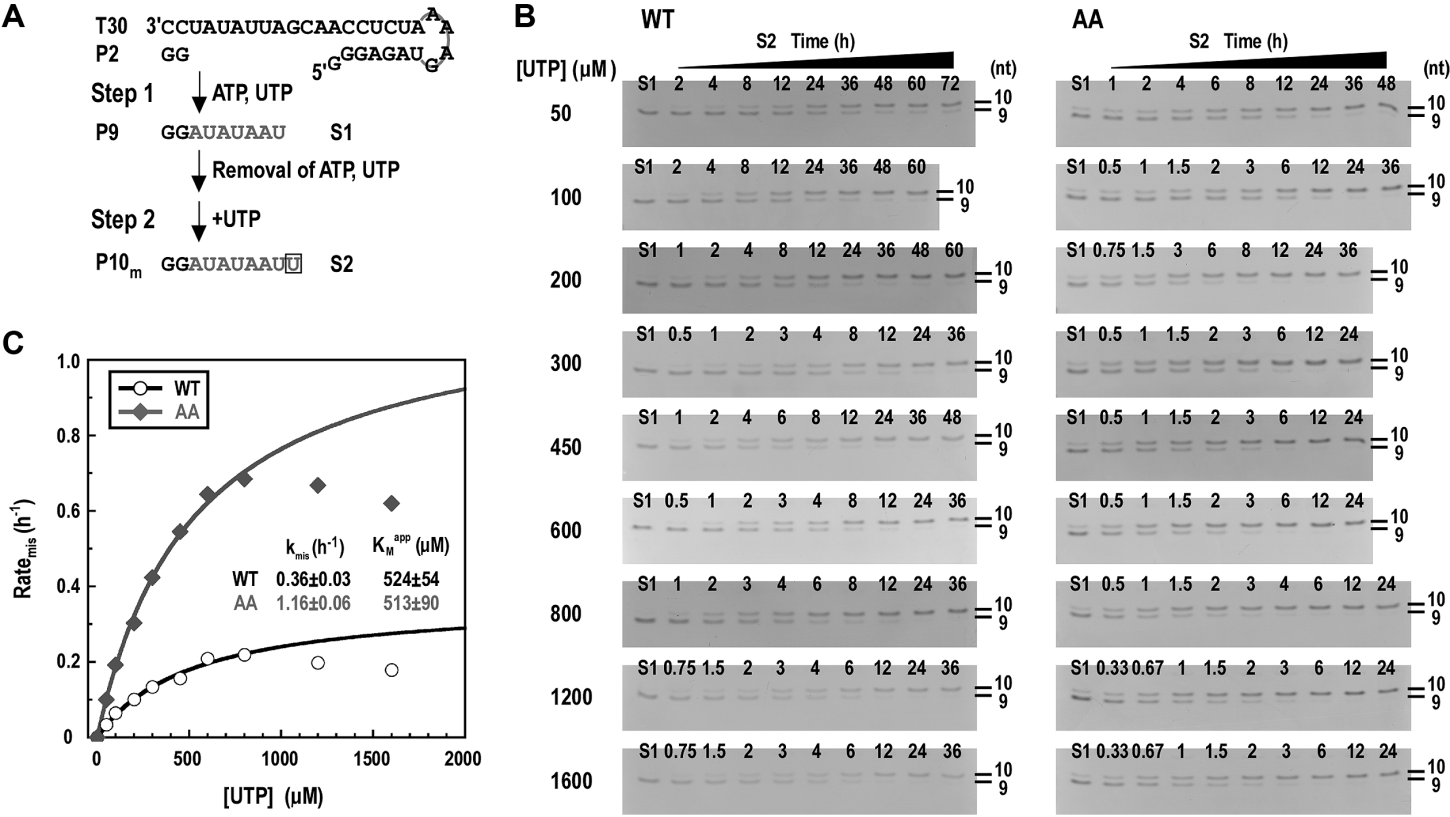
NS5B fidelity characterization using the snG:U_mis_ assays. (**A**) The reaction flow chart and products of the snG:U_mis_ assays. (**B** and **C**) Monitoring the snG:U_mis_ in a time course manner under a series of UTP concentrations for two representative NS5B constructs (WT and AA). The time course data under each UTP concentration were used to calculate the misincorporation rate (rate_mis_), and rate_mis_ values were then fit to the Michaelis–Menten equation to generate the *K*_M_^app^ and *k*_mis_ values. The data points circled by dashed oval were not included in the fitting due to the inhibitory effect observed under the highest two UTP concentrations.

To further dissect the mechanism of fidelity modulation by the NTD, we determined the misincorporation rate constants (*k*_mis_) and the apparent Michaelis constants (*K*_M_^app^) for the WT and AA mutant using a single-nucleotide G:U_mis_ (snG:U_mis_) assays. Similar to the P9 to P10 conversion assays, the snG:U_mis_ assays was designed in a two-step format (Figure 6A). The UMP misincorporation reactions converting P9 to P10_m_ were performed at 30 °C under a series of UTP concentrations after the removal of the originally supplied ATP/UTP (Figure 6A and B, step 2). When UTP was supplied at very high concentrations (e.g. 1200 and 1600 μM), an inhibitory effect was observed for both NS5B constructs (Figure 6C). Therefore the misincorporation rates (rate_mis_) measured under these concentrations were not used in the Michaelis–Menten curve fitting routines. The *k*_mis_ value of the AA mutant is about 3.2-fold of that of the WT (1.16 h^−1^ versus 0.36 h^−1^), while the *K*_M_^app^ values for WT and AA mutant are very much consistent (524 μM versus 513 μM) (Figure 6C). These data together suggest that the fidelity modulation by NTD is likely not through initial NTP binding but related to subsequent events leading to active site closure and the phosphoryl transfer reaction. This is consistent with the structural observation that NTD interacts with the RdRP palm in the vicinity of the active site closure-modulating motifs A and D but not the motif F containing ring finger involved in NTP binding.

## DISCUSSION

### A unique mode of fidelity modulation and implications for RNA virus vaccine development

The current work unravels the functional relationship between the pestivirus NS5B NTD and its natural fusion partner RdRP. As a small and unique globular domain, the NTD is connected to the RdRP through a flexible five-residue linker that could allow global NS5B conformational switching leading to disengagement of NTD and RdRP, as suggested by the two crystallographic conformational states identified in this study. Very interestingly, the intra-molecular interactions between the NTD and RdRP appear to have important roles in maintaining the RdRP fidelity at relatively high level. Structurally, the fidelity modulation by NTD likely achieved through its close proximity, in the closed conformation, to motifs A and D that are the only regions undergoing backbone movement during the RdRP active site closure (Figure 3A and B) (23). When the NTD–RdRP intra-molecular interactions were perturbed or absent, the RdRP fidelity was apparently impaired as suggested by data from all tested types of *in vitro* misincorporation assays. The pestivirus NS5B therefore represents a unique RdRP that may modulate its fidelity level through the interaction from a naturally fused domain. Although NTD–RdRP disengagement, a situation mimicked by the crystallographic open conformation state in a crystal lattice or the N-91 construct in solution, results in fidelity reduction, engaged NTD–RdRP with perturbation from point mutations can achieve similar level of fidelity reduction. As suggested by the two open conformation crystal structures (C-672_AA and C-672_E472A), the NTD–RdRP disengagement might occur in solution. In order to find out which conformational state is dominant in solution, we performed gel filtration chromatography and trypsin proteolysis analyses for the WT and representative mutants. In the chromatography analysis, the crystallographic open conformation C-672_AA and the C-672, or the AA mutant and the WT, had consistent retention volumes (Supplementary Figure S5A–C). In the trypsin proteolysis analysis, the WT and AA mutant had largely consistent proteolytic profiles (Supplementary Figure S5D and E). If compared to WT, the N-91 mutant obviously had characteristic proteolytic products, presumably related to the exposed RdRP palm surface due to the absence of the NTD (Supplementary Figure S5F). By carefully comparing the proteolytic profiles at representative time points for all three constructs, the AA mutant also produced a few proteolytic products that either had obviously different amount from the WT or were consistent with the characteristic products of the N-91. These observations suggest that though the closed conformation is dominant for the AA mutant in solution, the open conformation AA mutant likely exists in solution as a minor fraction. We propose that the pestivirus NS5B achieve its optimal fidelity level through the maintenance of native NTD–RdRP intra-molecular interactions. Alteration of these interactions, either by naturally occurring or engineered mutations or through NTD interactions with viral and host factors, can result in a change in fidelity level.

Fidelity adjustment mutagenesis based live attenuated vaccine design has been a valid rational approach to develop vaccines for RNA viruses (29,35,36). However, the majority of these attempts utilized fidelity modulation sites within the RdRP core and identifying optimal mutation sites has not been straightforward. Due to the unique fidelity regulation mechanism, the pestivirus NS5B may serve as an ideal system to testify and achieve fidelity alteration through the intra-molecular NTD–RdRP interface based mutagenesis design, and sites on both sides of the interface can be utilized for mutations.

### A comparison with the previously reported functional characterization of the pestivirus NTD

**Figure 7.**
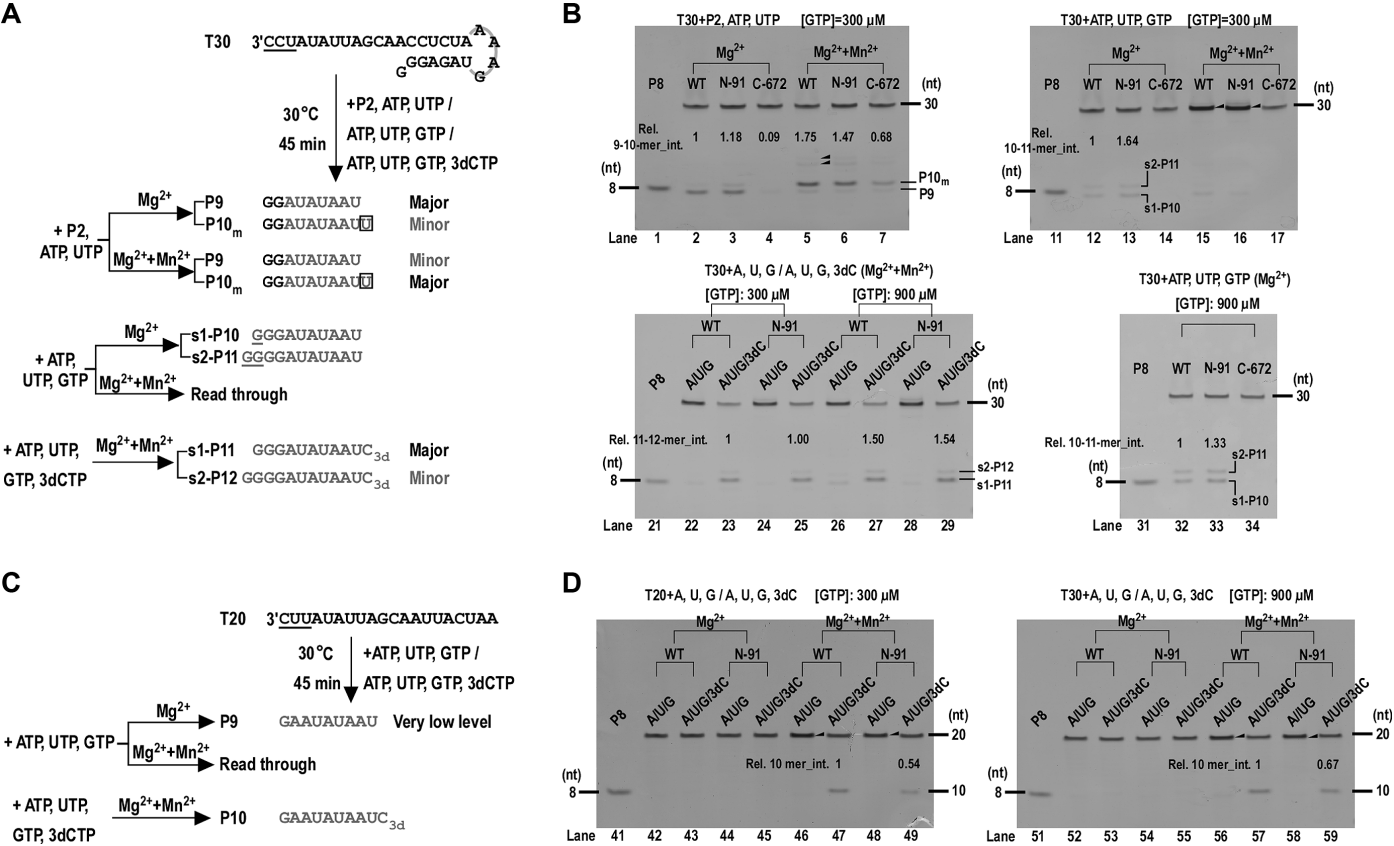
Mn^2+^ and the starting template sequence affected the overall synthesis activities of NS5B and the effect brought by NTD deletion. (**A**) Reaction flow-charts and product designations of the P2-based assays and the *de novo* (P2-free) assays both using T30 as the template. (**B**) Assessment of the impact on the NS5B synthesis by Mn^2+^ and the NTD deletion. In the P2-based assays the primary product was a 10-mer mismatch product (P10_m_) instead of the 9-mer correct product (P9) when ATP and UTP were supplied and 1 mM Mn^2+^ was supplemented. Longer products (solid triangle in the top left gel) likely derived from Mn^2+^-induced misincorporation were also evident. The WT and N-91 read through the template T30 (indicated by band intensity enhancement at the migration position of the template and the solid triangle in the top right gel) in the *de novo* assays when ATP, UTP and GTP were supplied and 1 mM Mn^2+^ was supplemented. (**C**) The reaction flow-chart and product designations of the *de novo* assays using a 20-nt template (T20) with a different starting template sequence from the T30. (**D**) Assessment of the WT and N-91 synthesis using the T20-based assays. The WT and N-91 had very low activities under Mn^2+^-free condition if compared to data of the T30-based assays. When 1 mM Mn^2+^ was supplemented, both the WT and N-91 read through (indicated by the solid triangles) the template T20 with ATP, UTP and GTP supplied. The reactions with ATP, UTP, GTP and 3′-deoxy-CTP (3dCTP) supplied were designed to largely inhibit the read-through activity and to allow a comparison of the synthesis levels of the WT and N-91 in the presence of Mn^2+^. In panels A and C, the starting trinucleotides of the T30 and T20 RNA are underlined for comparison.

Functional characterization of the NTD have been reported in both the BVDV and CSFV systems, largely by comparing the *in vitro* RdRP activities of the WT NS5B and their N-terminal truncated mutants (42,44,62). However, these studies focused on the overall *de novo* synthesis activities and the conclusions drawn by these studies have not been consistent. In a BVDV study using a 21-nt self-priming prohibited template derived from the 3′-terminal sequence of the viral minus strand RNA (starting template sequence: CAU), NS5B constructs with the N-terminal 38 and 81 residues deleted had 23% and 52% of *de novo* synthesis activities of the WT level with 1 mM Mn^2+^ present in the assay buffer (44). In a CSFV study using a long template derived from viral sequences (length and sequence sense not specified), NS5B constructs with the N-terminal 35 and 62 residues deleted had about 85% and 10% of the *de novo* synthesis activities of the WT level (62). In the recent CSFV study reporting the NTD-containing crystal structure, a 19-nt template very similar to the BVDV study (self-priming prohibited; starting template sequence: CAU) was used and an NS5B construct with the N-terminal 89 residues deleted produced 19% WT-level 19-mer products with 1 mM Mn^2+^ present in the assay buffer (42). However, a 20-mer likely arose from Mn^2+^-induced activities was the dominant product in this study. Since Mn^2+^ is known to facilitate the viral RdRP initiation and misincorporation (63) and the starting template sequence may also affect the initiation level, we performed comparative analysis of our T30/P2-based assays, the *de novo* (P2-free) T30 assays, and a type of *de novo* assays using a 20-nt template (T20) with a different starting sequence (CUU versus CCU in T30), using either our Mn^2+^ free assay condition or with 1 mM Mn^2+^ supplemented (Figure 7). In every assay type tested, the presence of Mn^2+^ resulted in obviously enhanced overall activities and pronounced misincorporation activities (Figure 7, compare lanes 2–4, 12–14, 42–45, 52–55 with lanes 5–7, 15–17, 46–49, 56–59). For the two P2-free assays with different starting template sequence under Mn^2+^-free conditions, the overall synthesis activities were very different. While the T30-based assays had moderate amount of products (Figure 7B, lanes 12 and 32), the product level of the T20-based assays was very low and barely detectable even under 900 mM initiating GTP concentration (Figure 7, lanes 42 and 52). We compared the activities of the WT and the N-91 and found that the N-91 had a 54–164% of the WT-level activities, no matter which assay types were tested or whether the Mn^2+^ was used. Collectively, these data confirmed that the Mn^2+^ and the starting template sequence can affect both the overall activity level and the effect brought by the NTD deletion. When Mn^2+^ was present, we did not observe obvious evidence of fidelity difference between the WT and the N-91. It is probable that in these assays and in the Mn^2+^-based assays in the literature, the fidelity effect brought by the NTD deletion identified in our P2-based Mn^2+^-free assays can be masked by the presence of Mn^2+^. Our data, in particular the data of the snG:U assays that specifically showed the fidelity modulation by the NTD–RdRP interface perturbation in the elongation phase (Figure 6), provide unambiguous evidence for the linkage between the NTD and the RdRP fidelity.

### The NTD–RdRP crosstalk of the pestiviurs NS5B contributes to the structural and functional diversity of viral RdRPs

The viral RdRPs have versatile global architecture beyond the conserved catalytic core comprising the palm, fingers and thumb. The *Picornaviridae* RdRP (e.g. poliovirus 3D^pol^) and the HCV NS5B represent the least complicated RdRP without fused domains beyond the RdRP core (11,12). The pestivirus NS5B represents RdRPs that have a small-size (∼100 residues) fused domain, while the flavivirus RdRP (e.g. JEV NS5) and the *Coronaviridae* RdRP (namely nsp12) represent RdRPs that have a medium-size (∼300–400 residues) fused domain/region (41,64). The *Bunyavirilaes* RdRP (namely L protein) represents RdRPs that have several fused domains/functional regions (65), while the *Orthomyxoviridae* RdRP complex (PA-PB1-PB2) represents RdRPs that function with intensive structural folding with other proteins (66). The diversity in RdRP global organization and its functional coupling to its fusion/folding partner(s) likely reflect the diversity in evolutionary origin of the viruses and virus-host co-evolution. Based on the best of our knowledge, the pestivirus NTD neither has high sequence homology nor high structural homology with proteins in any other systems. However, two structural implications likely support the evolutionary relationship between the pestivirus NS5B and the flavivirus NS5. As the first implication, the structures of the RdRP NE of pestiviruses and flaviviruses are highly analogous, although the NE sequences of the two virus genera are not obviously related (41). To date, no NE-like structures have been identified beyond these two viral genera. In amino acid sequence, pestivirus and flavivirus NEs are also similarly connecting the RdRP core and an N-terminal region: the NTD in pestivirus or the MTase in flavivirus. Although about three times the size of the pestivirus NTD, the MTase is also a single-domain module and also adopts an α/β fold with a seven-strand β-sheet flanked by several α-helices (67). Although quite speculative, the pestiviurs NTD and the flavivirus MTase might come from the same origin and have achieved its current function through divergent evolution.

## DATA AVAILABILITY

The atomic coordinates and structure factors for the reported crystal structures of the WT CSFV NS5B and its variants C-682, C-672, C-672_AA, Y471A, C-672_Y471A (form 1), C-672_Y471A (form 2) and C-672_E472A have been deposited in the Protein Data Bank under accession numbers 5YF5, 5YF6, 5YF7, 5YF8, 6AE4, 6AE5, 6AE6 and 6AE7 respectively.

## Supplementary Material

Supplementary Data
