# Supplementary material for: A unique intra-molecular fidelity-modulating mechanism identified in a viral RNA-dependent RNA polymerase
Source: Nucleic Acids Res. 2018 Sep 20;46(20):10840–54. doi: 10.1093/nar/gky848 (PMC6237809; doi:10.1093/nar/gky848)
Supplement: Supplementary Data [file gky848_supplemental_files.pdf]

Supplementary Data for revised manuscript

(tracking # NAR-02346-H-2018) entitled

"A unique intra-molecular fidelity-modulating mechanism identified in a viral RNA-dependent RNA polymerase"

by Weichi Liu [1,2], Xiaoling Shi [1,2], and Peng Gong [1,#]

1. Key Laboratory of Special Pathogens and Biosafety, Wuhan Institute of Virology, Chinese Academy of Sciences, Wuhan, Hubei, 430071, China;

2. University of Chinese Academy of Sciences, Beijing, 100049, China.

# Correspondence to Peng Gong: gongpeng@wh.iov.cn

Running title: Identification of a cis-acting polymerase fidelity factor

Supplementary Figures and Figure Legends

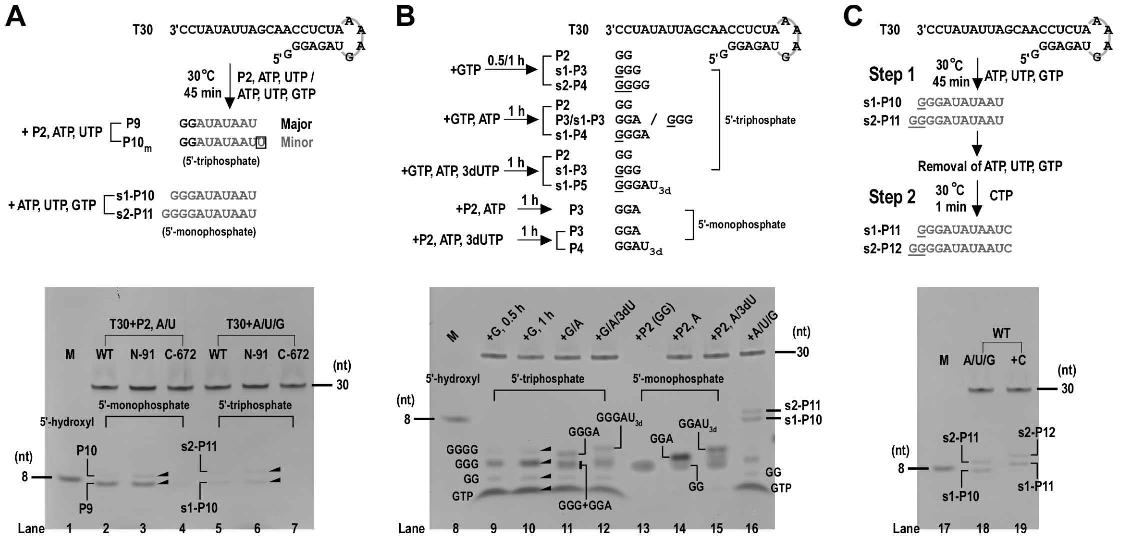

Figure S1. A comparison of the NS5B synthesis activities using the T30-based assays in the presence and absence of the GG dinucleotide (P2). **A)** A comparison of the WT NS5B and its N-/C- terminal truncated forms. The 8-nt marker (M; lane 1), the P2-derived products P9/P10 (lanes 2-4), and the products obtained in the P2-free assays (lanes 5-7) have hydroxyl, monophosphate, and triphosphate at the 5′-end, respectively. The different nature of the RNA 5′-end affected the migration of RNA. The WT and N-91 had lower activities in the P2-free assays than in the P2-derived assays, and the C-672 had very low activities in both assays. When ATP, UTP and GTP were supplied in the P2-free assays, the 10-nt (s1-P10) and 11-nt (s2-P11) products were produced instead of the expected 9 nt product. **B)** The 10-nt and 11-nt products generated in the P2-free assays were likely a result of slippage synthesis [1]. When GTP was provided as the only NTP substrates, in addition to the expected GG product, products of GGG (slippage mode 1 or s1) and GGGG (slippage mode 2 or s2) were evident in the P2-free assay, likely due to template-product slippage. Accordingly, the unexpected 10-nt and 11-nt products were then given the name of s1-P10 and s2-P11, respectively. **C)** A single nucleotide extension assay to confirm

the designation of the s1-P10 and s2-P11 products. The s1-P10 and s2-P11 produced by WT NS5B in the first step were rapidly extended to generate the s1-P11 and s2-P12 in the presence of the incoming CTP, suggesting that the s1-P10 and s2-P11 were produced due to the initial template-product slippage. In order to increase the amount of product for better observation, the GTP concentration in assays of B and C was increased to 900 μM, and the GTP concentration in assay A and all other NTPs were supplied at 300 μM each. The gel thickness was 1.5 mm in panel B for better visualization of GTP and dinucleotides. In regular 0.75 mm gels, GTP was non-detectable and dinucleotides were barely detectable likely due to fast diffusion of their low molecular weights. The P2-free assays were carried out same as the P2-derived assays except that P2 was not included in the reaction.

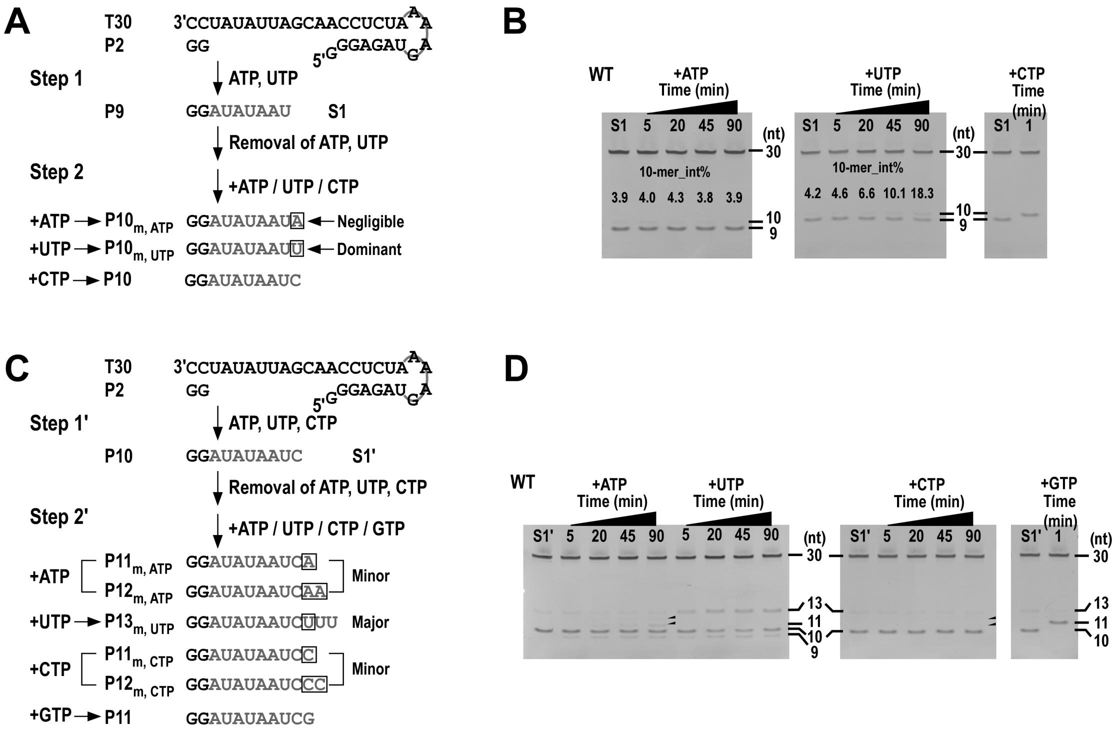

Figure S2. The evidences of G:U_{mis} and C:U_{mis} events. **A)** The confirmatory assay flow chart for the G:U_{mis} event. The boxed letters correspond to the misincorporated nucleotides. **B)** In the G:U_{mis} confirmatory assay, the ATP and UTP were removed after the 9 mer product (P9)-containing EC was formed in the first step. ATP, UTP, or CTP was then supplied as the only NTP substrate at 300 μM to monitor further elongation events. The fraction of the 10-mer product (P10) intensity (int) ($P10_{int}/(P9_{int}+P10_{int})$) values were calculated. The results indicated that P10 was hardly generated when only ATP was provided, gradually accumulated with time when only UTP was provided, and was very rapidly produced in the presence of the correct substrate CTP. These data suggest that the UMP misincorporation is the dominant misincorporation event in the G:U_{mis} assay for generating the $P10_m$ product when ATP and UTP were supplied as the only NTP substrates (Fig. 4 a and b). **C)** The confirmatory assay flow chart for the C:U_{mis} event. The boxed letters correspond to the misincorporated nucleotides. **D)** In the C:U_{mis} confirmatory assay, the ATP, UTP, and CTP were removed after the 10 mer product (P10)-containing EC was

formed in the first step. ATP, UTP, CTP, or GTP was then supplied as the only NTP substrate at 300 μM to monitor further elongation events. The data suggest that AMP, UMP, and CMP can be incorporated in the 10-mer to 11-mer misincorporation event, but the C:U_{mis} was much faster than the C:A_{mis} and C:C_{mis}. Therefore the 13-mer product ($P13_m$) is mainly derived from a C:U_{mis} event followed by two regular UMP incorporation events when ATP, UTP, CTP were supplied as the only NTP substrates in the C:U_{mis} assay (Fig. 4, c and d).

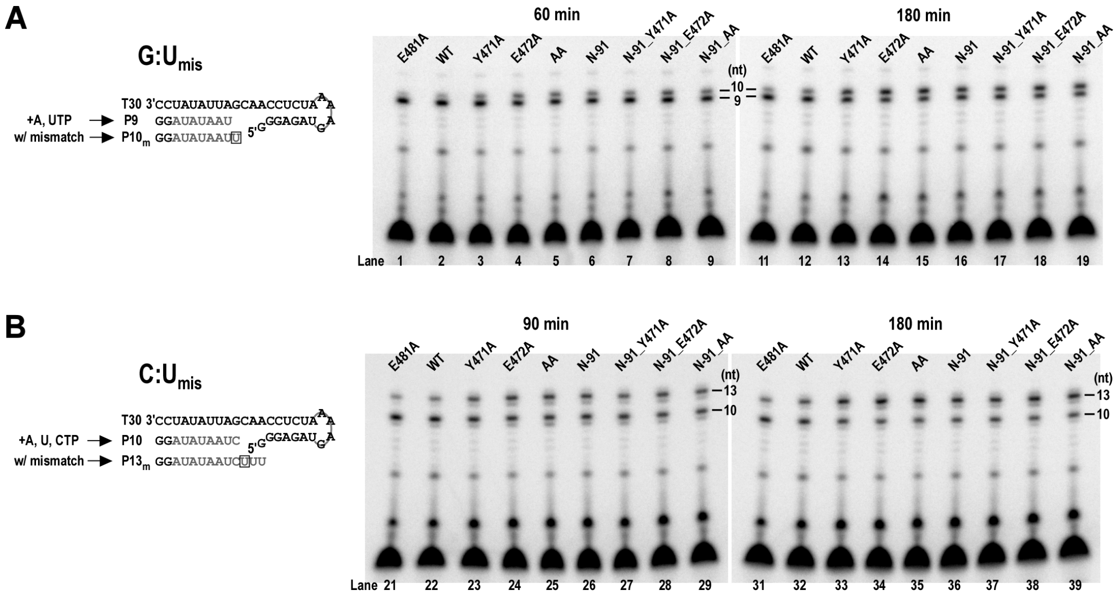

Figure S3. Supplementary data of NS5B fidelity characterization using the G:U_{mis} and C:U_{mis} assays. **A-B)** Representative gel sets for G:U_{mis} (A) and C:U_{mis} (B) assays corresponding to the column chart in Figure 4, b and d, respectively.

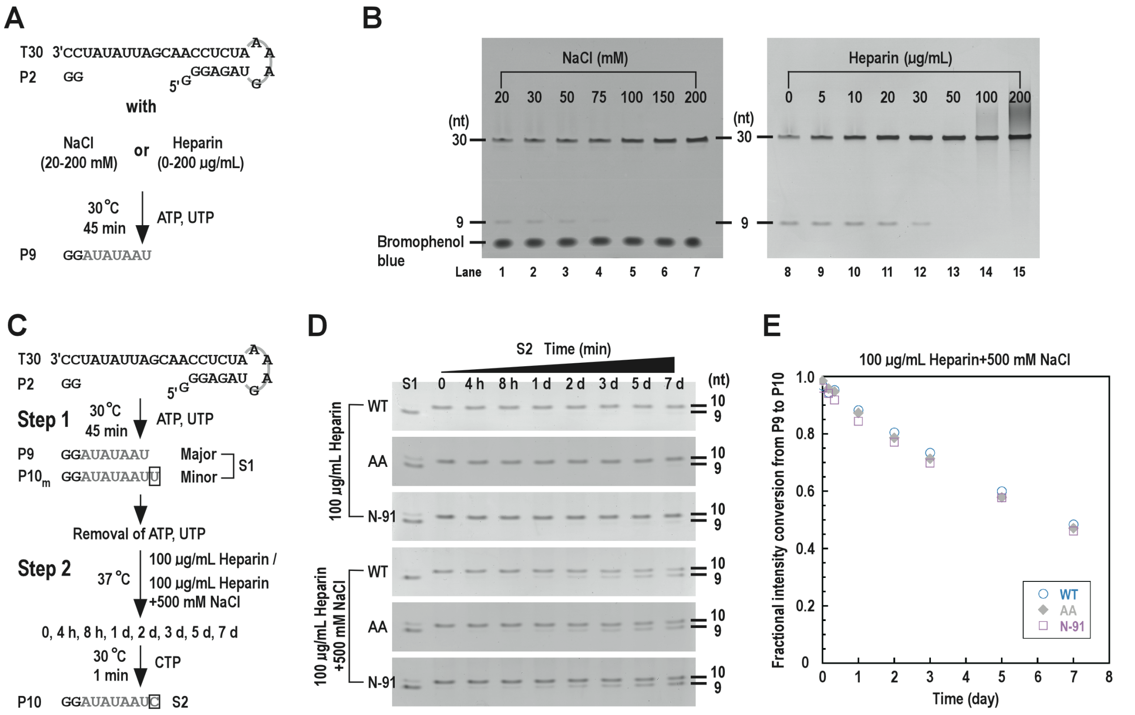

Figure S4. Supplementary data in assessing the stability of P9-containing complex. **A-B)** Reaction flow chart (A) and data (B) for the NaCl and heparin inhibitory tests on P2-driven P9 formation. When the NaCl concentration was 100 mM or higher, or heparin concentration was 50 μg/mL or higher, the P9 product was not detected. **C-D)** The reaction flow chart (C), the PAGE analyses (D), and quantification (E) for heparin/NaCl challenge tests of the P9-containing complex stability. The P9-containing complexes formed by different NS5B constructs were incubated under either 100 μg/mL heparin or 100 μg/mL heparin combined with 500 mM NaCl for different time period (up to 7 days) before the fraction of the complex survived the incubation was estimated by the fractional intensity conversion from P9 to P10 ($[P10_{int} - P10_{m,int}]/[P9_{int} + P10_{int} - P10_{m,int}]$, see details in main text Materials and Methods) plotted in panel E (only for the combined challenge). The majority of complexes formed by WT, AA, and N-91 constructs were able to complete the P9 to P10 conversion even after the 7-day challenge by 100 μg/mL heparin, while about 45% of the complexes survived the combined challenged after the 7 days. No significant

differences were observed among the complexes by different NS5B constructs tested, suggesting that the complex stability were not affected by the perturbation (AA) or removal (N-91) of the NTD-RdRP intra-molecular interactions.

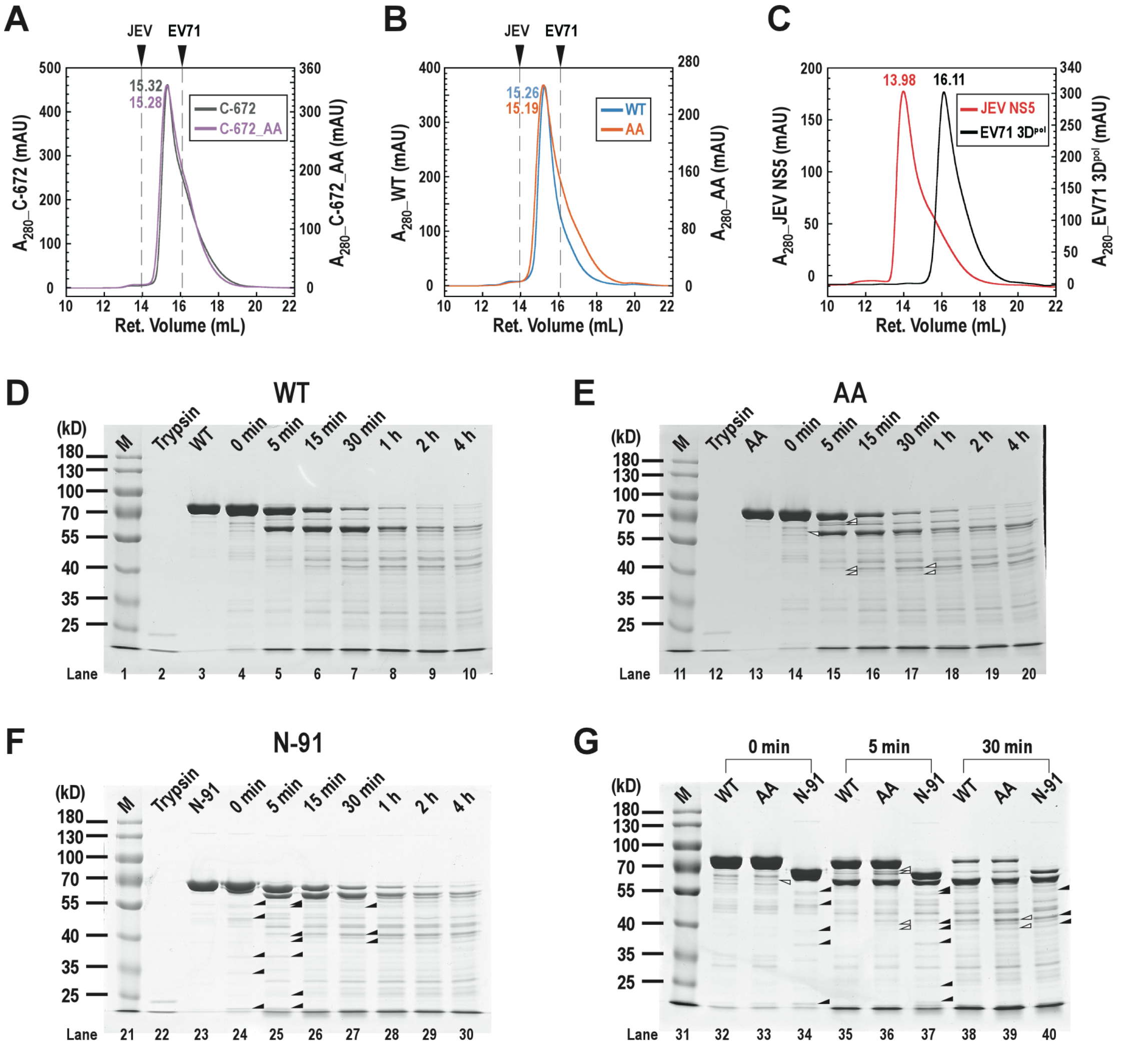

Figure S5. The gel filtration chromatography and trypsin proteolysis analyses of representative NS5B constructs. **A-C)** The gel filtration chromatograms of four CSFV NS5B constructs (WT/AA [81 kD] in panel A; C-672/C-672_AA [78 kD] in panel B), and two other viral RdRPs (JEV NS5 [104 kD] and enterovirus 71 [EV71] $3D^{pol}$ [52 kD] in panel C) obtained using a Superdex 200 Increase 10/300 GL column (GE Healthcare). The black triangles and dashed lines indicate the retention volumes of JEV NS5 and EV71 $3D^{pol}$, respectively. The curves of the absorbance at 280 nm (A_{280}) were normalized for comparison. **D-G)** The trypsin proteolysis assays of three CSFV NS5B constructs (WT in panel D; AA in panel E, N-91 in panel F, and three representative proteolysis time points of WT, AA and N-91 in panel G) with the

NS5B and trypsin at 50:1 (wt. to wt.). Characteristic proteolytic products of the N-91 or products with higher intensity than those of the WT are labeled by solid triangles. Proteolytic products of the AA that are either not present in the WT data or with different intensities are labeled by empty triangles (upward/downward: higher/lower intensity than that of the WT). The samples loaded in the gel of panel-G and the corresponding samples loaded in gels of panels D-F were from the same reaction but were loaded separately. In panels D-F, the coomassie-stained SDS-PAGE gels were shown in greyscale-mode by converting from the original RGB-mode without any brightness/contrast adjustment.

Supplementary Reference

1. Martin, C.T., Muller, D.K. & Coleman, J.E. Processivity in early stages of transcription by T7 RNA polymerase. *Biochemistry* **27**, 3966-74 (1988).

Supplementary Table

Table S1. X-ray diffraction data collection and structure refinement statistics of four NS5B constructs with point mutations.

PDB - construct<br>Global conf.	6AE4 - Y471A<br>"Closed"	6AE5 - C-672 Y471A<br>"Closed"	6AE6 - C-672 Y471A<br>"Closed"	6AE7 - C-672 E472A<br>"Open"
**Data collection**[1]				
Space group	$P\ 4_3\ 2_1\ 2$	$P\ 4_3\ 2_1\ 2$	$P\ 3_2\ 2\ 1$	$I\ 4_1\ 2\ 2$
Cell dimensions				
a, *b*, *c* (Å)	161.5, 161.5, 56.2	160.7, 160.7, 55.6	115.5, 115.5, 293.3	116.9, 116.9, 395.0
α, β, γ (°)	90, 90, 90	90, 90, 90	90, 90, 120	90, 90, 90
Resolution (Å)[2]	50.0-2.95 (3.06-2.95)	50.0-2.75 (2.85-2.75)	50.0-3.85 (3.99-3.85)	50.0-3.80 (4.09-3.80)
No. unique reflections	16,243	19,483	22,112	14,040
R_{merge}	0.148 (0.52)	0.118 (0.46)	0.146 (0.52)	0.144 (0.50)
R_{meas}	0.155 (0.54)	0.123 (0.48)	0.157 (0.56)	0.151 (0.53)
$I / \sigma I$	16.7 (5.6)	23.0 (6.5)	15.7 (4.3)	19.8 (7.3)
Completeness (%)	99.9 (100.0)	100.0 (100.0)	99.8 (100.0)	99.8 (99.9)
Redundancy	11.3 (11.5)	12.8 (12.2)	7.9 (8.3)	11.2 (11.6)
**Refinement**				
Resolution (Å)	2.95	2.75	3.85	3.80
No. unique reflections	16,191	19,408	22,045	13,913
R_{work} / R_{free} [3] (%)	19.1 / 23.7	18.1 / 23.3	20.9 / 24.7	21.2 / 24.6
No. atoms				
Protein	4928	5066	9522	4807
Ligand/Ion/Water	/ / 25	/ / 55	28 / / 6	12 / /
B-factors				
Protein	41.8	52.3	95.3	104.7
Ligand/Ion/Water	/ / 36.9	/ / 44.0	94.4 / / 76.8	94.1 / /
R.m.s. deviations				
Bond lengths(Å)	0.009	0.008	0.003	0.003
Bond angles (°)	1.004	0.882	0.682	0.589
Ramachandran stat.[4]	91.1 / 8.7 / 0.2 / 0.0	91.7 / 7.7 / 0.5 / 0.0	91.6 / 8.2 / 0.0 / 0.2	85.2 / 14.3 / 0.2 / 0.4

[1] One crystal was used for data collection for each structure. The crystals of PDB 6AE4 and 6AE5 grew with a precipitant solution containing 0.1 M tris (pH 8.0) and 60% (vol./vol.) poly (propylene glycol) 400, while the crystals of PDB 6AE6 and 6AE7 grew with a precipitant solution containing 0.1 M lithium acetate, 0.1 M bis-tris (pH 6.0), and 20% (wt./vol.) SOKLAN CP42.

[2] Values in parentheses are for highest-resolution shell.

[3] 5% of data are taken for the R_{free} set, and the same R_{free} set is applied for all structures.

[4] Values are in percentage and are for most favored, additionally allowed, generously allowed, and disallowed regions in Ramachandran plots, respectively.
